# Facilitators of Multisector Collaboration for Delivering Cancer Control Interventions in Rural Communities: A Descriptive Qualitative Study

**DOI:** 10.5888/pcd19.210450

**Published:** 2022-08-11

**Authors:** Peg Allen, Callie Walsh-Bailey, Jean Hunleth, Bobbi J. Carothers, Ross C. Brownson

**Affiliations:** 1 Prevention Research Center, Brown School, Washington University in St Louis, St Louis, Missouri; 2 Alvin J. Siteman Cancer Center and Division of Public Health Sciences, Department of Surgery, Washington University School of Medicine, Washington University in St Louis, St Louis, Missouri; 3 Center for Public Health Systems Science, Brown School, Washington University in St Louis, St Louis, Missouri

## Abstract

**Purpose and Objectives:**

Multisector collaboration is a widely promoted strategy to increase equitable availability, access, and use of healthy foods, safe places for physical activity, social supports, and preventive health care services. Yet fewer studies and resources exist for collaboration among governmental and nongovernmental agencies to address public problems in rural areas, despite an excess burden of risk factors for cancer morbidity and mortality. We aimed to learn about cancer prevention activities and collaboration facilitators among rural informal interagency networks.

**Evaluation Methods:**

In 2020, researchers conducted semistructured interviews with staff from rural public health and social services agencies, community health centers, and extension offices. Agency staff were from 5 service areas across 27 rural counties in Missouri and Illinois with high poverty rates and excess cancer risks and mortality. We conducted a thematic analysis to code interview transcripts and identify key themes.

**Results:**

Exchanging information, cohosting annual or one-time events, and promoting other agencies’ services and programs were the most commonly described collaborative activities among the 32 participants interviewed. Participants indicated a desire to improve collaborations by writing more grants together to codevelop ongoing prevention programs and further share resources. Participants expressed needs to increase community outreach, improve referral systems, and expand screenings. We identified 5 facilitator themes: commitment to address community needs, mutual willingness to collaborate, long-standing relationships, smaller community structures, and necessity of leveraging limited resources. Challenges included lack of funding and time, long travel distances, competing priorities, difficulty replacing staff in remote communities, and jurisdictional boundaries. Although the COVID-19 pandemic further limited staff availability for collaboration, participants noted benefits of remote collaborative meetings.

**Implications for Public Health:**

Rural areas need consistent funding and other resources to support health-improving multisector initiatives. Existing strengths found in the rural underresourced areas can facilitate multisector collaborations for cancer prevention, including long-standing relationships, small community structures, and the need to leverage limited resources.

**Summary**

**What is already known about this topic?**

Multisector collaboration is key to public health practice. Recent reviews highlight well-funded collaborative initiatives to prevent chronic disease. Much less is known about rural collaborations.

**What is added by this report?**

This report highlights rural agency perspectives on multisector collaboration and identifies facilitators unique to rural interagency collaboration. This report provides insights on how rural agencies across sectors (eg, health, education, social services) maintained collaborative relationships and pivoted to continue health-promoting services during the COVID-19 pandemic.

**What are the implications for public health practice?**

Existing strengths found in rural areas, including long-standing relationships and approaches to leveraging limited resources, can facilitate multisector collaborations for cancer prevention and control. Consistent funding and resources across rural sectors are needed.

## Introduction

Increasing equitable availability of and access to healthy foods, safe places for physical activity, smoke-free environments, tobacco use cessation supports, and cancer screening requires multisector collaboration at multiple levels of society (1). Multisector collaboration, also called intersectoral or cross-sector collaboration, is the coordinated effort of governmental and nongovernmental agencies from multiple aspects of society to address public problems (1,2). At the local level, public health agencies, schools, social services, transportation, city planning and parks departments, food banks, nonprofits, and businesses are some of the key entities in addressing risk factors for cancer and other chronic diseases (1). To increase access to cancer screening, partnering public health and health care organizations can expand their collaborations to include transportation and social service sectors (3). Governmental public health agencies increasingly serve as conveners of multisector collaborations as an essential function and key component of broadened public health practice in Public Health 3.0 (4), though smaller local health departments (LHDs) have fewer resources to fulfill this role (4). Public Health 3.0 calls for a progression in public health goals to address complex societal issues that affect health to ensure every resident has equitable access to health-promoting services and programs and opportunities for well-being. Such complex problems cannot be resolved through the efforts of any single sector. Multisector collaboration can help diversify resources, promote collective action, and better address social determinants of health (4).

Partly as a result of long-standing underinvestment and systematic disinvestment, rural US counties experience an excess burden of risk factors for cancer and cancer mortality but have limited resources for addressing these issues (5–7). Poverty (7), physical inactivity (6), obesity (6,7), tobacco use (6,7), heavy alcohol use (6), and food insecurity (7) are higher in US nonmetro areas than in metro areas. Furthermore, rural residents often have poorer health care access and are less likely to use preventive services than urban residents (7,8). Substantial barriers to multisector collaboration exist in rural areas, such as geographic distances and limited resources (eg, funding, staff). Less is known about facilitators that enable or support rural multisector collaboration for cancer prevention and control, as rural collaborations are less well studied (9–11).

## Purpose and Objectives

The purpose of this pragmatic qualitative study was to describe collaborative cancer prevention activities and facilitators of informal multisector interagency collaborations among rural agencies in 5 low-income service areas across 27 counties in rural southernmost Missouri and Illinois. These data informed the development of a network survey to characterize rural collaboration networks (reported elsewhere) (12). Our study aimed to address the following evaluation questions: 1) What types of agencies collaborate to advance cancer prevention and control in low-income rural communities? 2) What types of activities do rural informal networks collaborate on to prevent and control cancer? 3) Which evidence-based interventions in tobacco and alcohol control, obesity prevention, physical activity, healthy eating, human papilloma virus (HPV) vaccination, and promotion of cancer screening do rural informal networks collaborate on? and 4) What factors facilitate or impede informal multisector collaboration for cancer prevention and control?

## Intervention Approach

LHDs, hospitals, and other health and social service agencies commonly collaborate to conduct community health assessments and develop and implement community health improvement plans (CHIPs) (13,14). Through the community health assessment process, community agencies review surveillance data and provide input to prioritize the many competing community health needs. The CHIP is an action plan that guides how the selected priorities will be addressed. In underresourced rural areas, interagency collaborations often operate informally to address CHIP priorities, chronic disease prevention, behavioral health issues such as opioid abuse, and social issues such as food insecurity. While CHIP prioritization of community issues provides a guide to collaborative activities, informal networks also face emerging community needs and the issue of balancing collective priorities with each agency’s mission and scope (15).

Informal collaborative networks connect governmental and nongovernmental agencies across sectors to address complex community issues (16–18). Such networks strive to improve implementation of interventions at multiple levels and settings. Informal networks often have weak or diffuse oversight (15,19). Instead of having a single funding source, informal networks combine resources that have varying stipulations for program delivery (15,19). Although informal networks are common in prevention, they are less well studied than formal grant-funded networks or policy networks (15), especially in rural areas (20).

## Evaluation Methods

This is a descriptive qualitative study (21,22) conducted in 2020 via individual key informant interviews with agency staff in governmental public health (LHDs), nongovernmental health care organizations (Federally Qualified Health Centers [FQHCs] and hospital systems serving rural areas), education (Extension services), and social service agencies. The study took place in rural southern Missouri and Illinois, an area where cancer-related risk factors and outcomes are poorer than US averages (23–25). The Washington University in St Louis Institutional Review Board provided human subjects approval as an exempt study.

### Setting

At the request of study team members from the Missouri Department of Health and Social Services (MO DHHS) and the Missouri Colorectal Cancer Steering Committee, we focused the study in the rural multicounty service areas of 4 FQHCs funded by the MO DHHS and the Centers for Disease Control and Prevention to use evidence-based interventions to increase access to and rates of colorectal cancer screening. Because colorectal cancer incidence and mortality was also high in neighboring counties in Illinois, we included a multicounty rural area in Illinois.

The 5 service areas each had 4 to 7 counties, for a total of 27 rural counties. County population sizes ranged from 4,249 to 78,324, with a median of 13,693 and a total population of 503,274 across the 27 counties (26). Poverty rates were higher and insurance levels lower than state and national averages. Across the 27 counties, the proportion of individuals living at or below the US federal poverty guidelines as of 2018 averaged 20.5%, compared with the Missouri, Illinois, and national averages of 14.2%, 13.1%, and 13.1% respectively (26). The percentage of individuals reporting any health insurance in 2018 averaged 88.6% across the 27 counties, compared with 90.3% in Missouri, 92.7% in Illinois, and 91.1% nationally (26). All-cancer mortality for 2014 through 2018 averaged 199.7 per 100,000 population across the 27 counties, compared with 170.0 in Missouri, 160.0 in Illinois, and 156.0 nationally (25). Behavioral risk factors for cancer (physical inactivity; tobacco use; low fruit and vegetable intake; lack of recent breast, cervical, or colorectal cancer screening) were all higher across the 27 rural counties compared with state and national values (23,24).

### Sampling approach

We took a data-informed purposive sampling approach in which we developed county-level risk profiles using risk behavior (23,24) and cancer mortality data (25), and LHD staff full-time equivalents as a proxy measure for public health capacity (27). We selected 1 county with high cancer risk and 1 county with low cancer risk within each of the 5 service areas in which to focus interviews. The rationale was that there may be more cancer control resources in higher risk counties but that greater health needs could strain public health capacity. We used a combination of purposive, convenience, and snowball sampling approaches to identify rural agency staff from the selected counties to invite to interview. In each service area, we started interview invitations with LHDs, FQHCs, and state university Extension offices. We then used snowball sampling to identify additional social service, behavioral health, and health care agency staff to interview.

### Interview guide

The semistructured interview guide was developed by the multidisciplinary study team and informed by prior research, published literature, and the socioecological framework, which emphasizes multiple layers of influence from individuals to intra-organizations to local community and larger sociopolitical factors. Although we did not pilot the interview guide with agency staff, we received input from a MO DHHS representative and solicited recommended changes from the first 3 interview participants. We iteratively refined the guide throughout the study to ensure that we used appropriate terminology in our questions and elicited the intended information. For interviews conducted beginning June 2020, we added questions to explore how the COVID-19 pandemic had affected intra-agency services, clients, and inter-agency collaboration. Those being interviewed received a copy of the interview guide via email before the interview. Introductory statements explained the interview purpose: to learn about collaborations for cancer prevention and control, including the topics of physical activity, healthy eating, tobacco control, HPV vaccination, and cancer screening. Initial questions asked about the service area, the participant’s role and years of experience, and intra-agency approaches to the listed topics. Table 1 lists the collaboration questions and prompts. As is appropriate with semistructured interviews, interviewers asked a core set of questions across interviews, while remaining flexible in probing for additional information (22).

**Table 1:** Interview Guide to Determine Facilitators and Challenges to Collaboration on Cancer Prevention and Control in Rural Areas, Missouri and Illinois, 2020

Topic	Questions
Background	To start off, how would you describe your organization’s service area? Can you tell me a little bit about your role in your organization? How long have you been in your current position? In this organization? How long have you worked in your field overall?
Agency cancer control efforts	What programs, if any, does your organization have that promote, recruit, or refer people for breast cancer screening? For cervical cancer screening? For colorectal cancer screening? Which cancer prevention topics are important to your organization? What does your agency do to promote physical activity, healthy eating, and tobacco control? Is there some new innovation or practice in your setting affecting uptake or use of evidence-based strategies that you’re excited about? If yes, please describe.
Partnering organizations	Which are the key organizations and groups in your service area that your organization works with around cancer prevention and promotion of cancer screening? Probe on collaborations on cancer screening, tobacco control, physical activity, healthy eating, obesity prevention, referral networks.
Collaborative activities	Please list the types of things your organization does with other organizations as you collaborate around cancer prevention and control. What kinds of tasks or activities do you do together?
Desired future collaborative activities	Can you tell me about tasks that you don’t currently work on with other organizations but that you would like to?
Facilitators	What factors facilitate the agency collaborations we’ve been talking about?
Challenges	What challenges do the agency collaborations you’re involved in face in addressing community needs in cancer prevention and control?
COVID-19	How have things changed in your agency since the coronavirus hit the US? How are things going? How has the coronavirus affected your organization? How has the coronavirus affected your day-to-day work? How has the coronavirus affected your agency collaborations and networks? How has it affected your organization’s ability to collaborate? How might your ongoing collaborations look different? Any collaborating with organizations you haven’t worked with previously to address your community’s health related needs? What strategies are your teams and collaborations using to overcome challenges encountered during COVID? Any workarounds you’ve found helpful? Any solutions to the challenges you’ve mentioned?

### Data collection and analysis

A study team member extended interview invitations by telephone and email. We conducted interviews in person in February to mid-March 2020 and by telephone in June through August 2020. We planned to conduct interviews in person so that interviewers would develop fuller appreciation of contextual issues, but only a small number of in-person interviews in 4 of the 5 service areas were conducted before pausing research in mid-March because of the COVID-19 pandemic. Each interview took 40 to 60 minutes to complete; participants were offered a $40 gift card in thanks for their time. We audio-recorded interviews and used rev.com to transcribe each verbatim.

We used a thematic analysis approach, incorporating inductive and deductive code development (28). Deductive code development was informed by the principles of Public Health 3.0, the Community Guide, and the socioecological framework (4). Two coauthors (P.A., C.W.B.) reviewed all transcripts, drafted a codebook, pilot-coded 2 transcripts, then met to refine the codebook and generate consensus. The same 2 coauthors then independently coded each transcript in NVivo 10 (QSR International) and met weekly to reach consensus on discrepancies. Pairs of coauthors then created code reports summarizing key themes and illustrative quotes within each domain (eg, preventive services, collaboration activities, collaboration facilitators and barriers), using methods described by Saldana (28). Because we found no differences in participant views by counties with high or low cancer risk, we combined analyses across all participants and service areas.

## Results

### Participant characteristics

We conducted 32 interviews with rural agency employees, 13 in person and 19 by telephone. The number of interviews in each service area ranged from 5 to 7 and were evenly distributed among high and low cancer risk areas. Of the 32 participants, 29 were women and 3 were men. On average, participants had worked in their profession a mean (SD) of 15.9 (10.6) years, in their current agency a mean (SD) of 9.7 (10.5) years, and in their current position a mean (SD) of 5.2 (7.3) years. Twelve participants were on the leadership team in their agency, 7 were program managers, and 13 were specialists such as quality coordinators, nurses, dietitians, or health educators. Eleven participants worked in an FQHC, 11 in an LHD, 4 in Extension, 3 in local hospital systems, 2 in social service agencies, and 1 in a behavioral health agency. Of the 9 people who were invited to be interviewed and declined, 6 were in clinical roles at FQHCs or hospitals and 3 were LHD directors.

### Collaborative activities for cancer prevention and detection

Interagency collaborative activities included activities such as annual or one-time events to increase awareness and promote HPV vaccination or cancer screening and shared grant writing and establishment of ongoing collaborations to increase access to and promote physical activity and healthy eating (Table 2). We identified 5 types of activities from participants’ descriptions of their actual collaborations: 1) exchanging information about agency services and updates; 2) cohosting, helping with, or promoting annual or one-time community events to increase awareness of and promote health and social services; 3) promoting other agencies’ ongoing services or programs; 4) developing or sustaining ongoing services and programs; and 5) codeveloping or sharing of resources.

**Table 2:** Types of Collaborative Activity for Cancer Prevention and Control in Rural Areas, Missouri and Illinois, 2020^a^

Activity type	Illustrative quote	Example
Exchange information	“Each partner will talk about what's going on in their agency, if they have any new initiatives, if they have any events coming up.” (FQHC3 clinical director)	Refer clients to partner agencies
	“They are a referral source for us and we are a referral source for them.” (LHD5 health educator)	
Cohost or help at awareness events	“We may get invited to participate in a back-to-school fair and talk to the kids while they get a sports physical, we can also talk to the parents about making sure that they’re current on their immunizations. Or we may go to a health fair, a senior fair, and talk to an older group of citizens about just making sure that they get their annual wellness checkup, those types of things.” (FQHC4 quality improvement coordinator)	Annual all-community agencies/free services day Early childhood resource fair Strollin’ Thru the Colon Back-to-School fair
Promote each other’s programs	“Whenever we’ve got anything that’s going on about the FIT program or anything else, we share that with them [behavioral health, social services] and they share it out with their clients too.” (LHD1 communications director)	Joint marketing Help recruit participants Share other agencies’ fliers
	“We help promote some of their classes.” (LHD3 director)	
Develop or sustain ongoing programs	“And we talk about the projects that we have, we plan. We talk about the best way to approach things. And then we have formal plans with tasks and timelines and we go over that and see where we are.” (Social Services2 director)	Coplan and co-implement Co-identify project sites Cowrite local ordinance
Develop or share resources, including staff	“The school approached us initially and said, ‘Hey, there’s this opportunity [for after school programs] that we’d like to work with you guys on’.” (FQHC4 administrator)	Joint grant writing Shared social services navigator staff across health agencies
	“And so when grants come up, we talk about those . . . who’s going to write for them, who’s going to do what pieces of it.” (LHD2 health educator)	

Abbreviations: FIT, fecal immunochemical test to detect blood in stool to screen for colorectal cancer; FQHC, Federally Qualified Health Center; LHD, local health department.

a The numbers in the descriptive quote identifiers denote different organizations for that organization type.

Participants felt their collaboration networks were particularly successful at exchanging information and promoting each other’s annual or one-time events and ongoing services and programs. Regularly (at least monthly) exchanging information was viewed as important for knowing where to refer clients for what, because availability of assistance for basic needs (eg, food, utilities) and other services changed frequently in these underresourced areas. Staff said they shared information about other agencies’ events or ongoing services with clients and encouraged them to participate. Participants discussed readily helping each other host and promote annual or one-time events through rural radio stations, posting notices, and encouraging clients to participate. Collaborative groups typically met monthly, usually in person, before COVID-19, but pivoted to bimonthly or quarterly remote meetings during the pandemic. Participants described codevelopment and implementation of grant-funded prevention programs in 2 of the 5 service areas. FQHC staff noted tangible benefits of their close relationships and memoranda of agreement for cross-referrals in 2 different service areas, while participants in other areas wanted more formal improved referral systems. Participants also shared desired future collaboration efforts, including expanding availability of low-cost colonoscopy, mammography, and other preventive services for those with financial strain (uninsured or underinsured, low income); increasing community outreach; more joint grant writing; and codeveloping and implementing ongoing cancer prevention programs.

### Rural collaboration facilitators

We identified 5 facilitator themes from analyses of the interview transcripts: commitment to address community needs, mutual willingness to collaborate, long-standing relationships, smaller community structures, and necessity of leveraging limited resources (Table 3).

**Table 3:** Facilitators and Challenges of Multisector Collaborative Activity for Cancer Prevention and Control in Rural Areas, Missouri and Illinois, 2020^a^

Category	Theme	Illustrative quote
Facilitators	Commitment to address community needs	“We all have a common goal, I mean that’s a big one. I mean, we all have that common goal that we want to help our community . . . we’re all working towards the same goal so that just makes it easier.” (LHD5 dietitian)
	Mutual willingness to collaborate	“Well, willingness on both ends. I want what’s best for the overall population as do they, and we don’t want patients to not get the medical care that they need due to a financial burden, or an educational burden, or transportation issues. So I think just willingness on both ends to provide the necessary care that patients need is what makes that successful.” (FQHC1 quality improvement coordinator)
	Long-standing relationships	“With every partnership is give and take. I know that we could ask the parks department to do something for us and they would do it for us if they can . . . so it’s a lot of give and take there that we always know that we can always rely on each other.” (LHD2 health educator)
		“This community is very unique from other communities that I’ve worked in. And it’s a long-standing collaborative, long before I came on scene.” (Social Services2 director)
	Smaller community structures	“And so I think being a smaller community helps because you have the opportunity to know more people one on one.” (Social Services5 navigator)
	Necessity to leverage limited resources	“The thing about [this area] is it’s so small and there are so few resources that you have to work together because there just isn’t anything. So, nobody cannot really be their own island due to competition, even though competition exists everywhere. It’s just you have to work together down here.” (LHD1 director)
Challenges	Lack of funding	“We are a poor county. There is just not a lot of funding, wellness operations . . . so anything that we can collaborate on, we have to figure out how to do it without funding, because again, we’re just a very rural, low population, and poor county.” (Extension4 nutrition specialist)
	Replacing staff in remote communities	“I think especially in the rural areas, you either have your people that stay for life or they kind of use it as a jumping off point and they move somewhere else or switch jobs. So keeping up with all the contacts can be difficult.” (Extension5 health specialist)
		“Hiring qualified staff to do a lot of these things, that’s another thing that’s very difficult. . . . They will see us as an entry job and then they will stay for a year or so and move on to the next rung on the ladder. So, that makes it difficult because we’re constantly training and retraining.” (LHD1 director)
	Limited staff time for prevention and outreach	“Busy schedules. Sometimes it’s hard to get everyone together in the same room because every agency has so many responsibilities and you’re going so many different directions.” (Extension4 nutrition specialist)
		“That might take a lot of infrastructure and set up, a staff member who could just help navigate some of those things.” (LHD2 director)
	Geographic distances, travel time	“There’s travel time and there’s all that, and again, every agency, I think, faces the same problem.” (LHD1 communications director)
		“And, especially for poor communities south of [town], it’s hard for them to get 3 hours north. And, it’s hard for them to find transportation to get here. . . . But then, they’re like, ‘Oh, we want to help,’ and then nothing ever comes from it.” (Hospital3 nurse manager)

Abbreviations: FQHC, Federally Qualified Health Center; LHD, local health department.

a The numbers in the descriptive quote identifiers denote different organizations for that organization type.

**Commitment to address community needs**. Participants said that agencies come together around issues important to the local communities. “I think every organization wants to find the needs of the community and then try to focus there” (Extension4 nutrition specialist). Partners found it helpful to have flexibility to choose which community needs to prioritize. And “I think people have personal family or personal stories . . . or something that has hit a prominent community member. And a lot of times that drives some of the collaboration” (Hospital3 nurse manager).

**Mutual willingness to collaborate**. Multiple participants described “willingness on both ends” (FQHC1 quality improvement [QI] coordinator) and “a lot of give and take” (LHD2 health educator) to help each other meet needs of individual clients and the population as a whole. “So we have a really wonderful collaboration in this community among the different partners. People are always willing to come to the table and work on projects and collaborate with each other” (Social Services2 director). “I think just being open to actually doing the collaborations, I think that’s huge” (FQHC4 QI coordinator). “What I love about our community is just that everybody’s so willing to want to battle this and help one another out and really come together and minimize the gaps that are in the services” (Behavioral Health3 director).

**Long-standing relationships**. As noted above, participants had been in their agencies a long time, nearly 10 years on average. They stated that they had established relationships with partnering agency staff by gathering in interagency groups, working with 1 to 2 other agencies on particular initiatives, and chatting when seeing each other informally in grocery stores, places of worship, and community or cross-county regional events. Cross-pollination by serving on multiple groups furthered the “long-standing relationships” (Hospital3 nurse manager). “We have good relationships with all 4 counties and their directors of their health departments. A few of them even sit on our board, so that helps us tremendously.” “I’ve been in this position for 15 years. They’re partners, but they’re also friends, too” (LHD2 health educator).

**Smaller community structures.** Smaller community structures made it easier to find, communicate, and get to know people in cross-sector agencies. “I mean with us being so rural it’s easy to contact people and we have a really good communication line with pretty much any organization out there that needs us. We’re easy to get ahold of. We’re easy to find” (FQHC5 community outreach coordinator). “And so I think being a smaller community helps because you have the opportunity to know more people one on one” (Social Services5 navigator).

**Necessity of leveraging limited resources.** Participants noted interagency collaboration was a necessity because each rural agency had limited staff, funding, and other resources. “I think necessity is a big one. I think it’s [collaboration is] a need for sure. I don’t think anybody can tackle any of these issues on their own specifically. It’s just like any other disease or issue out there, public health issue that we’ve got to try and tackle as a group” (FQHC4 administrator). “It’s [collaboration is] a way to leverage resources. And then we, as a group, we often contribute to each other financially, not big money, but small amounts for this or that, if somebody has a need or is working on a project, some of that type of thing goes on also” (Social Services2 director).

Several participants described facilitating strategies to keep partners engaged, including staying in contact and ensuring partners had concrete tasks to contribute to the group. “Staying on top of it. If you haven't heard from a partner in a while, you reach out, ‘Hey, what’s going on at your organization? Haven’t seen you in a while? What's your newest?’ Just that constant of staying in touch with people” (Social Services5 navigator).

### Rural collaboration challenges

Table 3 shows key challenges that participants noted: lack of funding, limited staff time for prevention and outreach, and long distances and travel time to in-person interagency meetings. “The geography is a big deal here because everything is so spread out and the funding is so thin that it is very difficult to work around that” (LHD1 director). Staff time or availability limitations for collaborative prevention activities included competing priorities, staff or organizational scope restrictions, and turnover in the most remote areas, given recruitment challenges, and time needed to train new staff and establish partnering relationships. Two participants mentioned the challenges of geographical boundaries (eg, inaccessible nearby resources in a larger town across a state line). Participants discussed rural disparities in income and health status and frustrations, such as limited fresh produce for food pantry clients, as underlying challenges that made collective impact a long-term goal toward which their networks were taking small steps.

The COVID-19 pandemic brought additional collaboration challenges, which partners met with ingenuity. Pandemic response efforts and staff reassignments greatly restricted staff availability to collaborate on ongoing cancer prevention and control programs. Staff furloughs or reduced hours disrupted collaborative efforts. Interagency meetings pivoted to remote gatherings, with distracting technical glitches at the beginning. Several participants noted challenges in maintaining relationships through the less frequent remote meetings, noting online interactions were of poorer quality than in-person meetings. However, participants identified several advantages of remote meetings, including not needing to travel to collaborate and the ability to share resources in real time (eg, via web conferencing chat features). Several staff found increasing brief phone check-ins between meetings helped maintain relationships without overburdening partners.

Statewide partners offered technical assistance and training on telehealth and advocated for infrastructure support. Collaborating agencies shared information to help partners continue services during the pandemic, for example sending funding announcements and making connections to laboratories offering free processing of fecal immunochemical tests to detect blood in stool for colorectal cancer screening. Partners codeveloped new strategies for messaging community members on safely preparing for clinic appointments and the importance of continuing routine chronic disease maintenance and cancer screening. Participants described newly created or enhanced collaborations to meet community needs during the pandemic. For example, senior centers, churches, and local governments began working together to distribute food boxes and deliver meals to address increased food insecurity.

## Implications for Public Health

We identified facilitators of informal and formal multisector collaborative activities for cancer prevention and control through semistructured interviews with public health, health care, education, and social service agency staff in 5 rural service areas with low-income populations. Collaborative activities included exchanging information so agencies could best refer clients to each other and codeveloping and sharing resources, including staff. Agencies also cohosted cancer prevention and detection awareness events and promoted each other’s services and programs. Some codeveloped programs to increase availability of and access to healthy foods, safe places for physical activity, and preventive health care services including cancer screening. Five themes emerged as aspects that facilitate interagency collaboration for cancer prevention in rural underresourced areas across public health, health care, behavioral health, social services, schools, county extension offices, and local governments: commitment to address community needs, willingness to collaborate, long-standing relationships, smaller community structures, and necessity of leveraging limited resources.

### Collaboration framework

Several multisector collaboration facilitators identified in this study align with Bryson and colleagues’ “Framework for Understanding Cross-Sector Collaborations” (2,29). Bryson and colleagues postulate that initial agreement on the problem helps agencies clarify their interest or stake and acknowledge agency and sector interdependence to co-create solutions. Long-standing relationships are important in the framework, as it is through the lens of pre-existing relationships and networks that partners assess trustworthiness and legitimacy of other partners and new collaborative efforts. Previous positive interactions enable coordination, whereas lack of prior relationships results in collaborations that start with small steps that do not require trust (2). Bryson and colleagues also present and compare several frameworks for multisector collaboration (29). Participants did not discuss several components in the frameworks, including collaborative groups’ governance structures or power dynamics, or planning for sustainability. Participants also said little about the political climate and other influences external to their service area beyond funding challenges and unhelpful insurance structures. It is unclear whether these omissions were due to limitations of the informal networks, the interview guide content, or participant hesitancy to discuss these topics. Another omission was the lack of framing of solutions with an equity focus, although participants did discuss disparities in risk factors, access, and health status as challenges. To address the complexities of rural health, we need to address health equity, place, and the historical, political, and social contexts that drive policy (7). To help ensure an equity focus, partners can integrate a health equity framework with 1 of the several frameworks for health-promoting multisector collaboration and ensure that marginalized groups and perspectives are represented in collaboration networks (7,29,30).

### Alignment with literature

Facilitators identified in this study align with facilitators of collaboration formulation that were identified in recent reviews of multisectoral collaborations for health promotion. In our study, participants discussed commitment to working together toward a common goal as a key facilitator, aligning with a recent systematic review of facilitators of multisector alliance effectiveness in public health, which found alliances deemed as synergistic all had a common goal and clear purpose (31). What participants in our study described as willingness to collaborate aligns with the positive partner motives reported in the review (31). Timm and colleagues found shared goals and geographic proximity may facilitate development of multisectoral physical activity networks (32). Stolp and colleagues found trust and relationship quality were related to effectiveness of collaborations (33). Long-standing relationships highlighted in this study parallel the partner resources of personal networks, connections, and reputations identified by others as facilitators (34). Facilitators from recent reviews, including leadership (33,34), communication (31,34), and length of collaboration (32,33), were also discussed in our interviews.

Related studies offer support for the facilitators identified here. Zahner and colleagues found that a sense of community, leadership, having a neutral convening agency, funders’ expectations of collaboration, and identifying a common purpose with clear goals supported multisector collaboration in 4 counties with improved health outcomes (35). Studies commonly found that working toward a shared goal facilitated collaborating across sectors (35,36). Authors described openness to collaborating as willingness to participate in multisector partnering (36). In one of the few recent rural studies we found, relationship building, trust building, and shared vision and goals led to a shared valuing of health, while addressing policy and environmental change was a challenge (37). A shared purpose and prior collaboration history facilitated multisector collaboration, while geographic distance and lack of time were barriers in another rural study (10). Shared valuing of health and a sense of community were among facilitators of successful multisector collaborations in rural Iowa (9).

### Limitations

This qualitative inquiry had a few limitations. First, it was not feasible to interview collaborators from all 27 counties and our sampling approach possibly missed agencies with important views to contribute. Second, the health sector was overrepresented in the sample, limiting the perspectives obtained. Third, the need to learn the “what” of interagency collaborations to develop collaboration network surveys limited the depth of “why” and “how” information gathered. Nevertheless, others can use the facilitator information gleaned to expand their collaborations with additional sectors. That we included informal and formal collaborations for any topic in cancer prevention and control is both a limitation in terms of depth and a strength in terms of breadth.

### Implications for policy and practice

Rural areas need consistent funding and other resources to support health-improving multisector initiatives and counteract the long history of disinvestment in US rural communities (7,10,37,38). Rural areas need increased funding for prevention programs and services in general as well as increased funding for formal multisector collaborations to collaboratively implement specific initiatives. Given limited resources in rural agencies for cancer control (5), understanding assets and service capacity available in organizations and leveraging resources across networks can avoid depletion of agencies’ resources or service capacity. Because rural communities vary greatly in their contexts and populations, it is essential that local voices lead or share decision-making in such initiatives, regardless of funding sources and academic research constraints (7). Meaningful community engagement will also shine light on enhancing and leveraging rural strengths to address health equity, instead of continuing the rural deficit narrative.

Participants spoke little about evidence-based prevention programs; this aligns with previous work that found staff from LHDs serving smaller populations were less likely to use evidence-based practices (39), especially those involving policy or environmental changes (36). Few evidence-based interventions were developed or initially implemented in rural settings, and adapting interventions is complex; thus, rural agencies need the flexibility, funding, technical assistance, and tools to thoughtfully adapt interventions and document adaptation processes (7). The Rural Health Information Hub offers tool kits on numerous topics to support rural evidence-based intervention planning, implementation, and evaluation (https://www.ruralhealthinfo.org/toolkits). Network mapping tools are useful for identifying gaps and strengthening multisector collaboration. The high social capital in some rural areas is a strength that agencies can support and enhance to help sustain collaborations (7).

Although participants did not directly mention sustainability, agencies must collaboratively plan for sustainability of co-implemented initiatives (9,31,34,35). Practitioners can use available tools to support planning for sustainability, such as the Program Sustainability Assessment Tool (https://sustaintool.org/psat/). Because geographic distance is a substantial barrier to rural collaboration, the increased use of remote meeting platforms can continue to reduce travel time to in-person meetings. Enhancing the infrastructure of remote meetings (eg, broadband access, electronic video platforms) can support sustainment of multisector collaboration and coordinated implementation. The national push to address social determinants of health to improve equitable availability of and access to prevention initiatives may bring needed resources to rural multisector collaborations, especially to address rural food insecurity and inadequate access to healthy foods. Remiker and colleagues offer an approach for area multisector planning and prioritizing for equity action (40). Existing strengths found in the rural underresourced areas in our study can facilitate multisector collaborations for cancer prevention, including commitment to address community needs, long-standing relationships, small community structures, openness to collaboration, and willingness to share limited resources.
